# Consumers’ food choices, understanding and perceptions in response to different front-of-pack nutrition labelling systems in Belgium: results from an online experimental study

**DOI:** 10.1186/s13690-020-00404-3

**Published:** 2020-04-03

**Authors:** Stefanie Vandevijvere, Marie Vermote, Manon Egnell, Pilar Galan, Zenobia Talati, Simone Pettigrew, Serge Hercberg, Chantal Julia

**Affiliations:** 1 Sciensano (Scientific Institute of Public Health), J. Wytsmanstraat 14, 1050 Brussel, Belgium; 2 INSERM (Institut National de la Santé et de la Recherche Médicale), Paris, France; 3 INRA (Institut National de la Recherche Agronomique), Paris, France; 4 The George Institute for Global Health, Sydney, Australia

**Keywords:** Front-of-pack nutrition labelling, Belgian consumers, Perception, Food choices, Understanding, Nutrition policy

## Abstract

**Background:**

Front-of-pack nutrition labels (FoPLs) are increasingly implemented by governments internationally to support consumers to make healthier food choices. Although the Nutri-Score FOPL has officially been implemented in Belgium since April 2019, no study has been conducted before its implementation to compare the effectiveness of different FOPLs.

**Methods:**

The aim of this study was to compare food choices, objective understanding and perceptions of Belgian consumers in response to five different FOPLs, currently implemented in different countries internationally, namely the Health Star Ratings (HSR), the Multiple Traffic Lights (MTL), Nutri-Score, Guideline Daily Amounts (GDA), and Warning symbols. During the summer 2019, 1007 Belgian consumers were recruited and randomized to one of the five different FOPLs. Through an online questionnaire they were asked to choose one of three different foods within each of three categories (pizzas, cakes, breakfast cereals), as well as rank those same three foods according to nutritional quality, in the condition without as well as with FOPL. In addition, various questions were asked on their perceptions in relation to the FOPL they were exposed to.

**Results:**

Perceptions of consumers were favorable for all FOPLs with no significant differences between the different FOPLs. There were no significant differences in food choices among the different FOPLs, but Nutri-Score performed best for ranking food products according to nutritional quality.

**Conclusions:**

While there were no significant differences among different FOPLs for food choices and perceptions, the Nutri-Score was the most effective FOPL in informing Belgian consumers of the nutritional quality of food products.

## Background

Front-of-pack nutrition labelling (FOPL) has been repeatedly recommended by the World Health Organization (WHO) as one of a suite of measures needed to improve population diets [11, 24]. The policy objectives of FOPL are generally twofold: (i) to provide interpretive information to consumers to inform healthier food choices; and (ii) to encourage the food industry to reformulate their products towards healthier options. While an increasing number of governments internationally implement FOPL schemes, there are important differences in the algorithms, graphic formats and the regulatory approaches (i.e. voluntary or mandatory), which may influence their impact on both consumer as well as food industry behaviours. Summary systems (i.e. the Health Star Ratings (HSR) and the Nutri-Score, implemented on a voluntary basis), warning labels (implemented on a mandatory basis) and Multiple Traffic Lights (MTL) (implemented either on a voluntary or mandatory basis) are the most commonly implemented FOPL systems by governments [20].

A variety of government-endorsed nutrient profile models generally underpin these FOPL [12]. The voluntary Nutri-Score FOPL system, which was first implemented in France, was approved for implementation by the Minister of Public Health in Belgium in August 2018 and has been officially implemented in Belgium since April 1st 2019. All five biggest food retailers and a few food manufacturers have since either started or committed to put the Nutri-Score on their own brand products. The Nutri-Score is calculated based on the energy, saturated fat, total sugar, sodium, and fruit, vegetable, nut and legume (FVNL) levels and, in some instances, the protein and fibre content. The Nutri-Score rates the nutrient content of packaged foods with five colours/letters from red (least healthy) to green (most healthy). However, while in France, a comprehensive series of studies were conducted to test the potential impact of Nutriscore on consumers’ choices ahead of its implementation [10], in Belgium no such studies have been conducted to date.

The aim of the present study was to assess consumers’ food choices, objective understanding and perceptions in response to different FOPL systems currently implemented in different countries in the world, in a Belgian sample of consumers using the questionnaire and methods of the FOP-ICE study, an international experimental study conducted previously to compare the effectiveness of various FoPLs in 12 countries [6]. The following FOPLs were included in the study: the Health Star Ratings (HSR), Multiple Traffic Lights (MTL), Nutri-Score, Guideline Daily Amounts (GDA), and Warning symbols.

## Methods

The methodology was approved by the Institutional Review Board of the French Institute for Health and Medical Research (IRB Inserm n°17–404) and the Curtin University Human Research Ethics Committee (approval reference: HRE2017–0760). The study protocol has been described in detail elsewhere: http://www.ANZCTR.org.au/ACTRN12618001221246.aspx.

### Subjects and study design

An international ISO accredited web panel provider (PureProfile) based in Australia, was used to recruit Belgian consumers. Quota sampling was used to obtain equal-sized groups for age (one-third of participants in each of the following age brackets: 18–30 years, 31–50 years, over 51 years), sex (50% women) and socioeconomic status (one-third of participants in each of the following categories: low (< 14,292€), medium (14,292€-28,800€), and high (> 28,800€) yearly household income. The online questionnaire included questions on demographic and socio-economic characteristics, such as sex, age, monthly household income and education level, as well as lifestyle (i.e. involvement in grocery shopping, self-reported diet quality and nutrition literacy). In addition, participants were asked how often they purchased the food product categories under investigation (pizzas, cakes, and breakfast cereals). The food categories pizzas, cakes and breakfast cereals were selected for testing in the study because they are frequently consumed in Belgium and the nutritional quality of products within those categories is sufficiently varied. For each food category, three mock packages of foods with distinct nutrient profiles (higher, intermediate, and lower nutritional quality) were created. A fictional brand “Stofer” was used in order to prevent interference with other factors (e.g. brand loyalty, habit, preference, …) during the study.

**Fig. 1:**
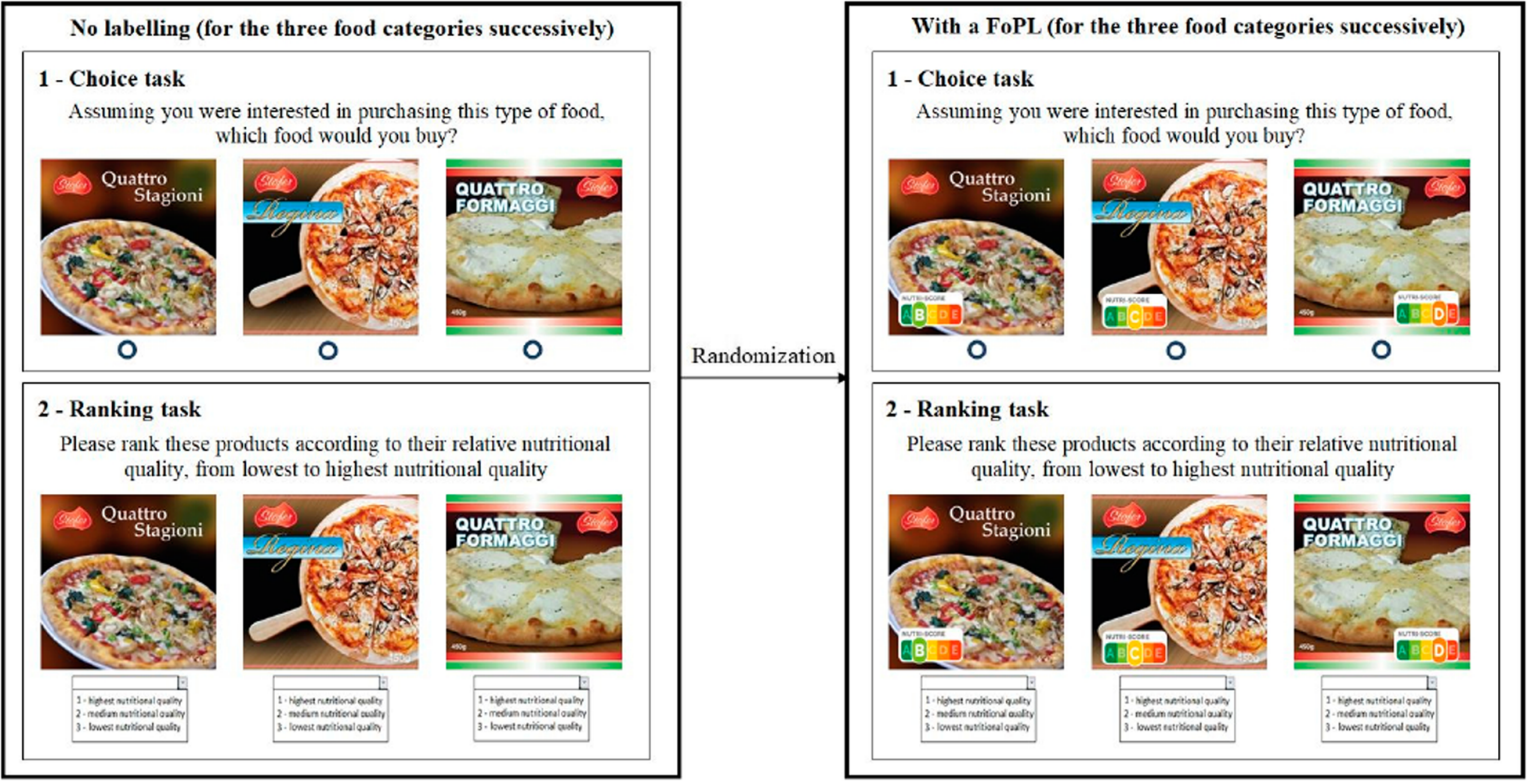
Example of procedure of the choice and ranking tasks for the pizza category

Five FoPLs were investigated, of which three nutrient-specific labels, namely [1] the Guideline Daily Amounts (GDA) displaying energy, sugar, (saturated) fat and salt content within a portion of a certain product and contributions to recommended daily amounts; [2] the Multiple Traffic Lights (MTL), displaying energy, (saturated) fat, sugar, and salt content of food in red, amber or green according to set thresholds; and [3] the Warning Symbols, displayed when the level of a given nutrient exceeds a specified threshold. The remaining FoPLs were summary systems, including [1] the Nutri-Score rating the overall nutritional content of packaged foods with five colours/letters from red/E (least healthy) to green/A (most healthy); and [2] the Health Star Ratings (HSR), using stars (from ½ to 5 stars) to show the nutritional profile of packaged foods (the more stars, the healthier the product). All FoPL variants were put on the package of the tested foods in the same place, covering approximately the same surface area. No other nutritional information or claims (e.g., organic certification; health and nutrition claims) were presented on the mock packages. Figure 1 presents an example of the set of tested pizza packages, with and without the FOPL.

Each participant served as their own control in this within-between subjects design. The control treatment was administered first for each part of the study explained below.

### Procedures

During the first part of the online questionnaire, participants provided sociodemographic, socio-economic and lifestyle information. The second part of the questionnaire included questions related to food choices, objective understanding and the perceptions in response to the assigned FoPL.

Firstly, participants were presented three different food products without FOPL for each of the three food categories and they had to select the item they would most likely purchase. The survey asked: “Assuming you were interested in purchasing this type of food, which food would you buy?” The option ‘I wouldn’t buy any of these products’ was also a possibility. This was followed by the task of ranking the same set of three products per food category (one set of three products for respectively pizzas, cakes and breakfast cereals) according to nutritional quality. Answer options for this task were: ‘1 - highest nutritional quality’, ‘2 – medium nutritional quality’, ‘3 - lowest nutritional quality’, and ‘I don’t know’. Secondly, participants were invited to repeat these same two tasks, but this time one of the five FoPLs were displayed on the packages, according to the randomisation conducted previously. Lastly, participants were presented several statements about their perception of the assigned FOPL. Statements included ‘Food companies should be able to choose whether they apply this label to their packaged foods’, ‘This label is confusing’, ‘It should be compulsory for this label to be shown on packaged food products’, ‘I like this label’, ‘This label does not stand out’, ‘This label is easy to understand’, ‘This label took too long to understand’, ‘This label provides me with the information I need’, and ‘I trust this label’, and had to be rated by participants on a 9-point Likert scale ranging from “strongly disagree” to “strongly agree”. To conclude the questionnaire, consumers were asked whether they had noticed the FoPL they were exposed to during their participation in the online survey.

### Data analysis

All analyses were performed in SAS 9.4. The statistical significance level was set at α = 0.05.

#### Food choices

For both label conditions (with and without FOPL), participants obtained 1 point when choosing the product with the lowest nutritional quality, 2 points when choosing the product with medium nutritional quality and 3 points when choosing the product with the highest nutritional quality. For each food category, a total score ranging between − 2 and + 2 points was calculated based on the difference in points between both label conditions (with and without FOPL). A total score was then obtained by summing the scores for each of the three food categories, resulting in a total score between − 6 and + 6 points.

Per food category and FOPL, percentages of participants improving or deteriorating their food choice between the no label and FoPL conditions was determined. In order to measure the association between the food category or total score for the food choice task and the FoPL type, ordinal logistic regression was conducted. Participants who did not select a product in either the no label or FOPL condition were excluded from the analysis. The models were adjusted for sex, age, household monthly income level, education level, involvement in grocery shopping, nutrition knowledge, self-reported diet quality and whether or not the FOPL was noticed during participation in the study. The GDA label was used as the reference of the models for the FoPL type categorical variable.

#### Objective understanding

Ranking products according to their nutritional quality was used to determine the objective understanding of the FoPL by the participants. A score of + 1 point was given per food category when the participants ranked all three products correctly. If at least one mistake was made − 1 points were attributed. When participants selected ‘I don’t know’ in either the no label or FOPL condition they received a score of 0 points and were excluded from the analysis.

For each food category, the difference in points between the two label conditions was calculated, resulting in a score ranging between − 2 and + 2 points. The sum of the scores of all three food categories resulted in a total score between − 6 and + 6 points. Per FoPL type and food category, the percentages of correct answers in both labelling conditions were calculated. In order to assess the association between the food category or total score for the objective understand task and the FoPL type, ordinal logistic regression model was used. The models were adjusted for sex, age, household monthly income level, education level, nutrition knowledge, involvement in grocery shopping, self-reported diet quality and whether or not the FOPL was noticed during participation in the study. The GDA label was used as reference of the models for the FoPL type categorical variable.

#### Perceptions

For each FOPL type, means and confidence intervals were calculated for each of the nine perception statements. A principal component analysis was conducted to calculate the contribution of the different statements to the overall perception of the different FoPLs. Dimensions, corresponding to a linear combination of statement variables, have an eigenvalue reflecting the total variance explained by the dimension. The number of retained dimensions was chosen to obtain a cumulative percentage of acceptable variance. Participants answering all perception questions the same were excluded from the analysis.

## Results

In total 1007 Belgian consumers participated in the online survey, of which 73% were responsible for grocery shopping, 23% reported a very or mostly unhealthy diet quality and 32% declared having no or little knowledge about nutrition. About 62% of participants reminded having seen the FoPL during the survey; these percentages were lowest for the warning symbols (40%) and the HSR (50%) (Table 1).

**Table 1:** Individual characteristics of the study sample from Belgium (*N* = 1007)

	N	%
**Sex**		
Men	505	50.2
Women	502	49.8
**Age, years**		
18–30	336	33.4
31–50	336	33.4
≥ 51	335	33.3
**Educational level**		
Primary education	55	5.5
Secondary education	328	32.6
Trade certificate	117	11.6
University, undergraduate degree	356	35.4
University, postgraduate degree	151	15.0
**Level of household monthly income**		
High	338	33.6
Medium	340	33.8
Low	329	32.7
**Responsible for grocery shopping**		
Yes	738	73.3
No	73	7.3
Share job equally	196	19.5
**Self-estimated diet quality**		
I eat a very unhealthy diet	17	1.7
I eat a mostly unhealthy diet	213	21.2
I eat a mostly healthy diet	634	63.0
I eat a very healthy diet	143	14.2
**Nutrition knowledge**		
I do not know anything about nutrition	31	3.1
I am not very knowledgeable about nutrition	287	28.5
I am somewhat knowledgeable about nutrition	634	63.0
I am very knowledgeable about nutrition	170	16.9
**Did you see the FOP label during the survey?**		
No	277	27.5
Unsure	110	10.9
Yes	620	61.6
**Participants who recalled seeing the FoPL they were exposed to**		
HSR	101	50.0
MTL	150	74.6
Nutri-Score	155	77.1
GDA label	133	65.8
Warning symbol	81	40.3

### Food choices

**Fig. 2:**
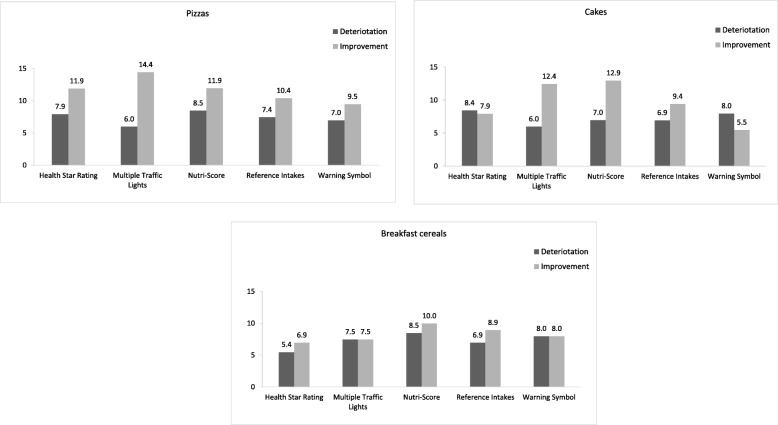
Percentage of participants having deteriorated or improved their food choices between the no label and FOPL condition

Between 54 and 68% of participants (dependent on the food category and FOPL type) did not change their choice between the no label and the FoPL conditions. Another 19 to 29% of participants (dependent on the food category and FOPL) did not select any product. Compared to the no label condition, the food choice differed significantly for the pizza (overall *p* value for Bowker disagreement test = 0.008) and cake (overall p value for Bowker disagreement test = 0.004) categories in the FoPL condition. Between 5.5 and 14.4% of participants (depending on the label and the food category) demonstrated an improvement in the nutritional quality of their choices while between 5.4 and 8.5% of participants demonstrated a deterioration (Fig. 2).

Overall, no significant associations between FoPL type and the change in nutritional quality of the food choices were found compared to the GDA label. Neither for each separate food category significant associations were found (Table 2).

**Table 2:** Associations between front-of-pack label type and change in nutritional quality of food choices by food category (*n* = 1007); using Guideline Daily Amounts (GDA) label as the reference of the models

Food category	N	HSR		MTL		Nutri-Score		Warning Symbols	
		OR (95% CI)	*p*	OR (95% CI)	*p*	OR (95% CI)	*p*	OR (95% CI)	*p*
All categories	917	1.001 [0.653–1.535]	0.9	1.324 [0.866–2.024]	0.2	1.056 [0.690–1.617]	0.8	1.004 [0.652–1.548]	0.9
Pizza	784	1.190 [0.700–2.021]	0.5	1.431 [0.854–2.399]	0.2	1.047 [0.624–1.759]	0.9	1.051 [0.620–1.784]	0.8
Cakes	785	0.873 [0.504–1.512]	0.6	1.499 [0.871–2.580]	0.1	1.491 [0.873–2.547]	0.1	0.998 [0.574–1.734]	0.9
Breakfast cereals	743	0.963 [0.547–1.698]	0.9	0.823 [0.468–1.448]	0.5	0.877 [0.497–1.549]	0.6	0.939 [0.533–1.656]	0.8

### Objective understanding

**Fig. 3:**
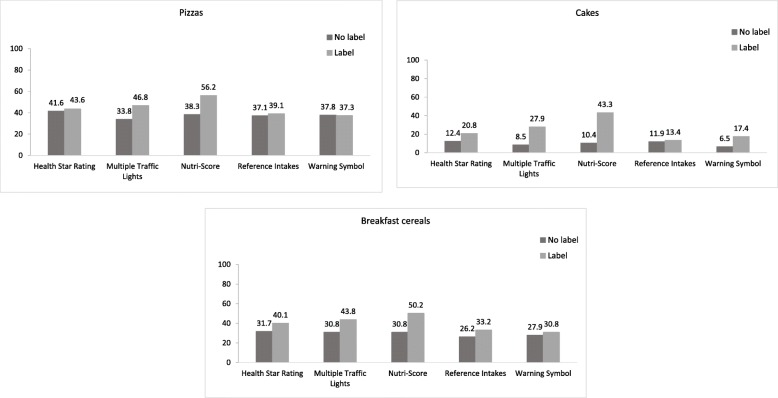
Percentage of correct answers for ranking foods by nutritional quality, by food category and FoPL

The percentage of correct responses improved for all five FoPLs compared with the no label situation, with the exception of the Warning Symbols in the pizza category where no differences were observed between both label conditions (Fig. 3). For all food categories, the largest increase in the percentage of correct responses between the no label and FOPL condition was observed for the Nutri-Score, with the highest increase found for the cake category.

For all separate food categories and for all food categories combined, significant improvements in the ability to correctly rank products according to their nutritional quality were observed for Nutri-Score FOPL compared to the GDA label (odds ratio (OR): all food categories = 3.167 [2.165–4.633] (*p* < 0.001); pizzas = 2.204 [1.263–3.847] (*p* < 0.05); cakes = 6.533 [3.649–11.696] (*p* < 0.001); breakfast cereals = 2.045 [1.167–3.581] (*p* < 0.05)). The MTL also significantly improved the ranking ability of the participants compared to the GDA label within all food categories (OR = 2.089 [1.432–3.048] (*p* < 0.001)) and the cake category (OR = 3.227 [1.775–5.868] (*p* < 0.001)), whereas the HSR and Warning Symbols were associated with significant improvements in the ability to correctly rank products compared to the GDA label in the cake category only (OR: HSR = 2.308 [1.234–4.317] (*p* < 0.05); Warning Symbols = 2.928 [1.585–5.409] (*p* < 0.001)) (Table 3). Nutri-Score was the label with the highest performance for all food categories, followed by the MTL.

**Table 3:** Associations between FoPL type and the ability to correctly rank products according to nutritional quality by food category (*N* = 1007); using Guideline Daily Amounts (GDA) label as the reference of the models

Food category	N	HSR		MTL		Nutri-Score		Warning Symbols	
		OR (95% CI)	*p*	OR (95% CI)	*p*	OR (95% CI)	*p*	OR (95% CI)	*p*
All categories	1007	1.369 [0.933–2.009]	0.1	2.089 [1.432–3.048]	< 0.001	3.167 [2.165–4.633]	< 0.001	1.246 [0.846–1.838]	0.3
Pizza	734	0.928 [0.519–1.659]	0.8	1.726 [0.986–3.021]	0.06	2.204 [1.263–3.847]	< 0.05	0.963 [0.543–1.711]	0.9
Cakes	753	2.308 [1.234–4.317]	< 0.05	3.227 [1.775–5.868]	< 0.001	6.533 [3.649–11.696]	< 0.001	2.928 [1.585–5.409]	< 0.001
Breakfast cereals	756	1.095 [0.609–1.970]	0.8	1.543 [0.877–2.713]	0.1	2.045 [1.167–3.581]	< 0.05	0.946 [0.524–1.707]	0.8

### Perceptions

**Fig. 4:**
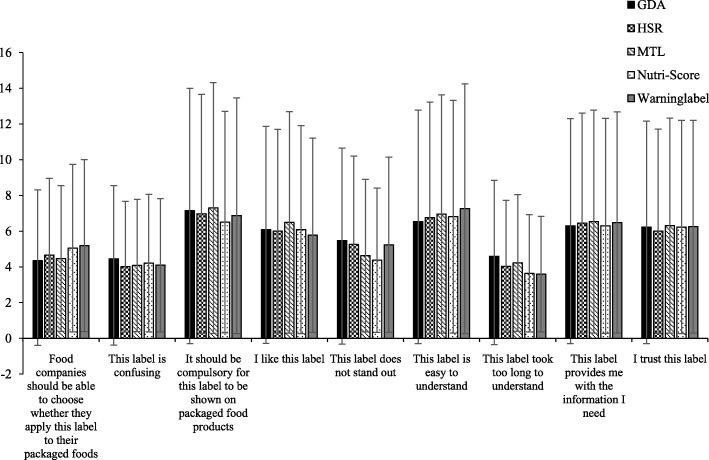
Averages scores with confidence intervals of perception statements by FoPL

Perceptions of consumers were generally favorable for all FOPLs with no important differences between the different FOPL types (Fig. 4). Two main dimensions were revealed by the principal component analysis explaining 35.9 and 25.4% of the total variance respectively. Table 4 displays the eigenvectors of the various statements on both dimensions. Items ‘I like this label’, ‘This label provides me with the information I need’, ‘I trust this label’ and ‘It should be compulsory for this label to be shown on packaged food products’ were most strongly (positively) correlated with the first dimension. Items such as ‘This label is confusing’, ‘This label took too long to understand’, ‘This label does not stand out’ and ‘Food companies should be able to choose whether they apply this label to their packaged foods’ were most strongly (positively) correlated with the second dimension (Table 4).

**Table 4:** Eigenvectors of active variables on the two dimensions from the principal component analysis

Perception Statements	Eigenvectors	
	Dimension 1	Dimension 2
Food companies should be able to choose whether they apply this label to their packaged foods	0.002	0.438
This label is confusing	−0.231	0.495
It should be compulsory for this label to be shown on packaged food products	0.381	0.047
I like this label	0.413	0.228
This label does not stand out	−0.102	0.419
This label is easy to understand	0.442	−0.005
This label took too long to understand	−0.184	0.528
This label provides me with the information I need	0.452	0.140
I trust this label	0.431	0.186

**Fig. 5:**
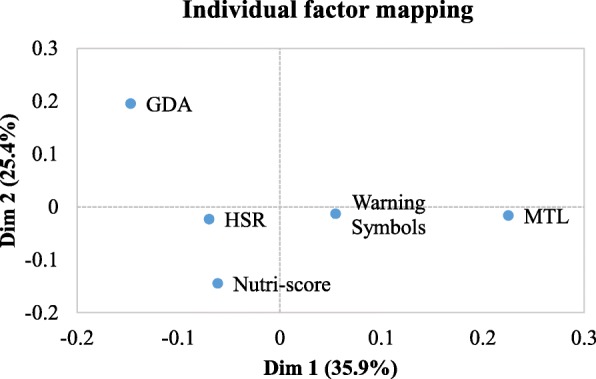
Projection of the FOPL labels on the two axes shown in a principal component analysis map

As the positioning of the different FoPLs on the principal component analysis map was between − 0.2 and 0.3, differences between the FOPLs for the two dimensions were relatively small (Fig. 5). The HSR and Nutri-Score appeared in opposition of the Warning Symbols and MTL on the first dimension, while the GDA label appeared to be opposite to all other FoPL on the second dimension. MTL turned out to be the most trusted and liked label by respondents, providing them the information they needed in contrast to the others.

The GDA label was found the most confusing, the least standing out and took the longest to understand by participants. The Nutriscore on the other hand was found to be the least confusing and the quickest to understand by participants. However, differences between the different labels were found to be small (Fig. 5).

## Discussion

Among a sample of Belgian consumers, there were no significant differences in food choices among the different FOPLs, but Nutri-Score performed best for ranking food products according to nutritional quality. Perceptions of consumers were favourable for all FOPLs with no significant differences between the different FOPLs. These results confirm that interpretive FOPL, and notably Nutri-Score, have greater potential than the GDA to support consumers to correctly rank the nutritional quality of foods and are similar to previous studies already conducted in several European countries [18] [7] and in Australia [1]. In France, additional research has been undertaken in relation to the impact of Nutri-Score on purchasing intentions using a randomized controlled trial in a virtual web-based supermarket [4] as well as in an experimental supermarket [9]. In the web-based supermarket, the intervention simulated shopping situations with front-of-pack nutrition labels affixed on food products. Around 12,000 participants were randomly assigned to one of five exposure conditions: GDA, MTL, Nutri-Score, Green Tick, or control (no front-of-pack exposure). The Nutri-Score significantly led to the highest overall nutritional quality of the shopping basket, followed by MTL and Green Tick, compared with the control, for all socio-economic groups. The Nutri-Score was also the only FOPL that led to significantly lower amounts in lipids, saturated fatty acids, and sodium of the shopping basket [4]. In the experimental supermarket, about 900 participants were recruited and distributed across three conditions: 1) control situation; 2) Application of the Nutri-Score on all breakfast cereals, sweet biscuits and appetizers; and 3) introduction of the Nutriscore accompanied by consumer information on use and understanding of the label. Significantly higher mean nutritional quality was found of sweet biscuits purchased in the intervention combining the label + education, but not for the other food categories [9].

A study using an experimental economy design compared in 691 participants, Nutri-Score, the HSR system, MTL, SENS (a format proposed by retailers) and a modified version of the GDA [3]. The nutritional quality of the shopping cart was improved by 9.3% for Nutri-Score, 6.6% for the HSR and 4.8% for MTL. Nutri-Score performed best in households with the lowest incomes. A large scale trial in the real world was performed in 60 supermarkets, 10 for each of four proposed labels (Nutri-Score, MTL, SENS and the GDA) and 20 controls [2]. Nutri-Score was associated with the largest improvement in the nutritional quality of the purchased items, followed by MTL and SENS. Moreover, the Nutri-Score was associated with an improvement in all subgroups of the population (in particular subjects buying discount brands), while other formats led to mixed results, with some subgroups deteriorating the nutritional quality of their purchased items.

Other, similar interpretive FOPL systems like the HSR in Australia/New Zealand have also shown to guide healthier food choices [15, 17], in particular among more nutrition-conscious shoppers [13]. In addition, MTL have been shown to encourage consumers towards healthier food choices, with red labels having more impact than green ones [16]. GDA labels however have been shown to have no or very limited impact on consumer food choices [8, 19]. More research is needed to investigate the real life impact of Nutri-Score and other FOP labels on food purchases and diets.

Some early evidence also suggests that interpretive labels may improve population diets through healthier product reformulation by the food industry. Adoption of the Choices nutrition logo in the Netherlands [21], the Health Check Program symbol in Canada [5], and the Pick the Tick logo in New Zealand [22] and Australia [23] and the Health Star Ratings in New Zealand and Australia [14] all led to reported reformulation of selected food products on the market. It is important for this aspect also to be taken into account when deliberating on the effectiveness of different FOP nutrition labels.

There are some strengths and limitations in our study. Strengths include the large number of participants, including participants from lower socio-economic groups, the investigation of a range of dimensions of FOPL effectiveness and the use of randomized approach. In addition, this is the first study on the potential effectiveness of Nutri-Score compared to other FOPLs in Belgium. Limitations include the quota sampling, the use of mock packages, fake brands and lack of access to nutritional information on the back of pack which differs from a real world setting.

## Conclusions

In conclusion, while there were no significant differences among different FOPLs for food choices and perceptions, Nutri-Score performed best for ranking products according to nutritional quality in a sample of Belgian consumers. Now that the Nutri-Score has been implemented voluntarily in Belgium and is starting to appear on the packages, it is critical to evaluate its impact on consumer purchases, industry reformulation and population diets.

## Data Availability

The datasets used and/or analyzed during the current study are available from the corresponding author on reasonable request.

## Abbreviations

FOPLs: Front-of-pack nutrition labels
GDA: Guideline Daily Amounts
HSR: Health Star Ratings
MTL: Multiple Traffic Lights
WHO: World Health Organization
